# Scaling-Stimulated Salivary Antioxidant Changes and Oral-Health Behavior in an Evaluation of Periodontal Treatment Outcomes

**DOI:** 10.1155/2014/814671

**Published:** 2014-11-03

**Authors:** Po-Sheng Yang, Wei-Chen Huang, Shyuan-Yow Chen, Chien-Hsun Chen, Chang-Yu Lee, Che-Tong Lin, Yung-Kai Huang

**Affiliations:** ^1^Department of Surgery, Mackay Memorial Hospital, Mackay Medical College, Taipei 104, Taiwan; ^2^School of Nutrition and Health Sciences, College of Public Health and Nutrition, Taipei Medical University, Taipei 110, Taiwan; ^3^Dental Department, Cathay General Hospital, Taipei 106, Taiwan; ^4^School of Oral Hygiene, College of Oral Medicine, Taipei Medical University, 250 Wu-Hsing Street, Taipei 110, Taiwan; ^5^Division of Periodontics, Department of Dentistry, Taipei Medical University Hospital, Taipei Medical University, Taipei 110, Taiwan; ^6^School of Dentistry, College of Oral Medicine, Taipei Medical University, Taipei 110, Taiwan; ^7^Division of Prosthodontics, Department of Dentistry, Taipei Medical University Hospital, Taipei 110, Taiwan

## Abstract

*Aim*. Our goal was to investigate associations among scaling-stimulated changes in salivary antioxidants, oral-health-related behaviors and attitudes, and periodontal treatment outcomes.* Materials and Methods*. Thirty periodontitis patients with at least 6 pockets with pocket depths of >5 mm and more than 16 functional teeth were enrolled in the study. Patients were divided into three groups: an abandoned group (AB group), a nonprogress outcome group (NP group), and an effective treatment group (ET group). Nonstimulated saliva was collected before and after scaling were received to determine superoxide dismutase (SOD) and the total antioxidant capacity (TAOC).* Results*. Salivary SOD following scaling significantly increased from 83.09 to 194.30 U/g protein in patients who had irregular dental visit patterns (<1 visit per year). After scaling, the TAOC was significantly higher in patients who had regular dental visits than in patients who had irregular dental visits (3.52 versus 0.70 mmole/g protein, *P* < 0.01). The scaling-stimulated increase in SOD was related to a higher severity of periodontitis in the NP group, while the scaling-stimulated increase in the TAOC was inversely related to the severity of periodontitis in the AB group.* Conclusions*. These results demonstrate the importance of scaling-stimulated salivary antioxidants as prognostic biomarkers of periodontal treatment.

## 1. Introduction

Periodontal disease is a serious public health problem worldwide. Prevalence rates of periodontitis among people aged 35~44 years are 54% in Taiwan and 47% in the United States [1, 2]. Periodontitis severe enough to result in tooth loss is found in 5%~15% of most populations around the world [3]. A meta-analysis by Lagervall et al. showed that periodontal disease and tooth loss are also related to systemic health issues such as cardiovascular disease, diabetes, and rheumatoid disease [4]. Epidemiologic and experimental evidence has identified risk factors for periodontal disease including microbiology (macrobiotic/periodontal pathogens), lifestyle (smoking and alcohol use), psychosocial factors, chronic diseases (diabetes and hypertension), and genetic factors [5]. There is increasing evidence of an association between poor oral hygiene and clinically important medical conditions of periodontal disease [6]. It is thus important for clinicians to examine factors in addition to those traditionally studied to increase the efficacy of periodontal disease treatment, management, and prevention.

Periodontitis is a chronic inflammatory disease caused by components of the indigenous oral microbiota and host inflammatory-immunologic responses [7]. The host works to maintain a condition of homeostasis in the internal environment, while the immune system wards off diseases by providing resistance against foreign organisms [8]. Periodontal tissue destruction occurs when homeostasis is thrown off by imbalances caused when foreign organisms induce oxidative and inflammatory injury [9]. Microbial interactions can cause oxidative stress from the activity of excessive reactive oxygen species (ROS) or a deficiency of antioxidants or from activating transcription factors that lead to proinflammatory states. This oxidative stress may be the most pertinent factor relating to periodontal tissue damage [10].

Oxidative stress is also an important factor in many disease conditions. Superoxides are converted into hydrogen peroxide and singlet oxygen by superoxide dismutase (SOD) within biological tissues. [11]. The total antioxidant capacity (TAOC) was therefore developed to reduce the costly and time-consuming task of measuring individual antioxidant species [12]. Kim et al. demonstrated that SOD activity in periodontitis patients was lower than that of control subjects at each time point during clinical treatment; on the other hand, the salivary TAOC was higher in control subjects than in patients with severe chronic periodontitis who had received scaling and root planing therapy [13]. A recent study reported that SOD activity significantly decreased in response to periodontal treatment, but the TAOC significantly increased in response to periodontal treatment [14].

Effective plaque control is the most important issue in preventing and controlling periodontal disease. While poor oral health is mainly related to periodontal disease [6], supragingival plaque can be eliminated by regular use of personal oral-hygiene equipment such as toothbrushes and fluoridated toothpastes [15]. One study showed that self-efficacious beliefs about oral hygiene are related to an increased tooth-brushing frequency, which is in turn associated with a better oral-health status [16]. Plaque control (scaling) and patient education (tooth-brushing instructions) are primary procedures for treating periodontitis. Ultrasonic scaling is an efficient way to remove calculus below the gum line [17]. Ultrasound activates a cell growth signaling pathway [18] and also stimulates cell secretion-related proteins to maintain or improve cell function [19]. We hypothesized that whole-body redox homeostasis is influenced by oral-health behaviors and attitudes and sequentially results in changes in salivary antioxidants after scaling stimulation or effective treatment in periodontitis patients. The specific aim of this study was to investigate associations among scaling-stimulated changes in salivary antioxidants, oral-health behaviors and attitudes, and periodontal treatment outcomes.

## 2. Methods

### 2.1. Subjects and Clinical Data Collection

**Figure 1:**
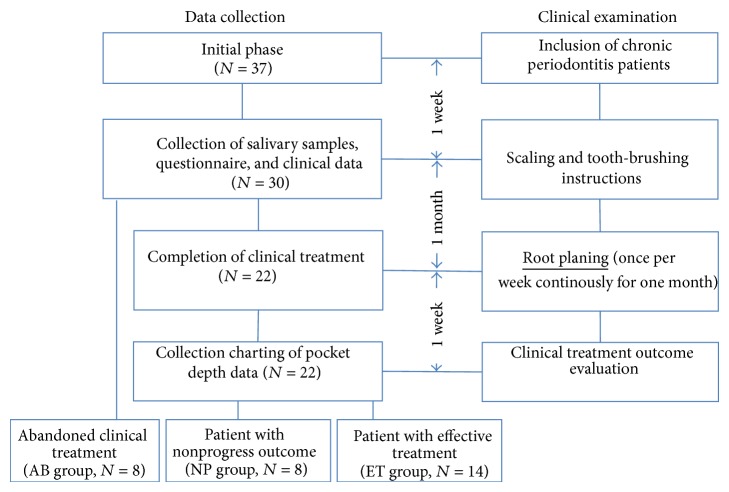
Data collection and clinical examination protocol scheme.

Data collection and clinical examination protocols are shown in Figure 1. Periodontal diagnostic criteria were based on the classification scheme of the American Academy of Periodontology [20]. Thirty-seven systemically healthy patients with chronic periodontitis (at least 6 pockets with pocket depths of >5 mm and more than 16 functional teeth) were included in the initial phase at the Cathay General Hospital Dental Department over a 6-month period (October 2011 to March 2012). Thirty patients consented to provide their saliva for this study. The study was approved by the Cathay General Hospital Institutional Review Board. Before conducting interviews and collecting samples, informed consent was obtained in writing from all subjects. The study complied with the World Medical Association* Declaration of Helsinki*.

A clinical periodontal examination was consistently performed by the same trained dental clinicians (Wei-Chen Huang, Shyuan-Yow Chen, and Chien-Hsun Chen,) to eliminate interexaminer variability. A patient received a periodontal examination and treatment from the same dental clinician. Pocket depth (PD) was evaluated as the distance between the gingival margin and the bottom of the sulcus/pocket and was assessed at six sites. Salivary samples were collected and clinical measurements performed at an appointment 1 week after patients had received scaling and tooth-brushing instructions. After scaling, patients underwent four root planing sessions during the next month (once per week). Eight patients did not complete this treatment and were defined as the abandoned (AB) group. Twenty-two patients completed clinical treatment, and their chart data of PDs were collected to evaluate the clinical outcomes of periodontitis treatment. Patients were defined as an effective treatment group (ET group, *n* = 14) when differences in 1~3 mm PD percentages increased or differences in 7~9 mm PD percentages decreased during the initial clinical treatment and after completing 4 weeks of clinical treatment. The remaining 8 patients were defined as a nonprogress outcome group (NP group, *n* = 8).

### 2.2. Questionnaire and Interview

Experienced assistants conducted a standardized personal interview based on a structured questionnaire. Information obtained from the interview included socioeconomic and demographic characteristics, lifestyle, oral-health behaviors, and oral-health-related attitudes. There were 9 items of oral-health attitudes including the following. (1) Do you agree that oral health is less important than systemic health? (2) Do you agree that dental calculus can be removed by brushing? (3) Do you consider your mouth to be healthy? (4) Do you believe that your teeth do not cause problems? (5) Do you believe that smoking may have an adverse effect on oral health? (6) Do you believe that dental calculus is caused by bacteria? (7) Do you believe that periodontal disease is a problem around a tooth? (8) Do you believe that scaling is an effective treatment for periodontal disease? And (9) do you believe that antibiotics are an effective treatment for periodontal disease? For the first four questions, “no” scored 1 point, “yes” scored −1 point, and “I do not know” scored 0 points. For questions 5~9, “yes” scored 1 point, “no” scored −1 point, and “I do not know” scored 0 points. The sum of all 9 items was used as the oral-health attitudes score.

### 2.3. Saliva Preparation and Antioxidant Determination

Nonstimulated saliva was collected before and after scaling during the patient's clinical examination the first week. A saliva specimen was collected with sterilized gauze pieces (20 × 20 mm), which were placed in the buccal and sublingual areas and recovered by centrifugation (1000 rpm, 3 min). More than 2 mL of unstimulated saliva was collected in the tube. The tubes (containing 2 mL saliva) were combined with 20 *μ*L protease inhibitor cocktail (Roche, Manheim, Germany) and centrifuged at 2000 g for 15 min. After centrifugation, the supernatant was separated and placed in a 1.5 mL Eppendorf tube. Tubes were stored at −20°C and were analyzed within 2 months. SOD activity was determined using commercial kits (Ransod, Randox Laboratories, Crumlin, UK). The TAOC was determined using the Total Antioxidant Status Liquid Stable kit (Fortress Diagnostics, Antrim, UK). SOD and the TAOC were determined with a Synergy H1 microplate reader (Biotek, Winooski, VT, USA) according to the manufacturer's instructions. In order to quantify changes in salivary antioxidants before and after scaling, differences were calculated by subtracting prescaling salivary antioxidant levels from postscaling salivary antioxidant levels.

### 2.4. Statistical Analysis

Data were analyzed using SAS 9.3 software (SAS, Cary, NC, USA). Because salivary SOD and TAOC in this study were not normally distributed, a nonparametric test was used for the data analysis. Data for comparisons of demographic characteristics, baseline clinical data, oral-health behaviors, and oral-hygiene attitude scores among the AB, NP, and ET groups were analyzed with Fisher's exact test. Differences between continuous parameters among groups were analyzed with a Kruskal-Wallis test. The Wilcoxon signed-rank test was used to analyze differences in SOD and TAOC values in the same subject before and after scaling. The Mann-Whitney *U* test was used to compare SOD and TAOC differences between two independent groups (stratified by oral-health attitudes or behaviors). Probability levels of <0.05 were used as criteria for significance.

## 3. Results

Demographic characteristics and oral-health behavior and oral-health attitude scores did not differ among the AB, NP, and ET groups (Table 1). There were no statistical differences between pre- and postscaling in any group. In this study, salivary antioxidants (SOD and the TAOC) exhibited no statistical differences among the three groups. The mean differences in SOD were −60.69, 37.64, and 14.91 U/g protein in the AB, NP, and ET groups, respectively. The mean differences in TAOC were −11.07, 1.58, and 0.52 mmole/g protein in the AB, ET, and NP groups, respectively. SOD and the TAOC exhibited no statistical differences between pre- and postscaling in any group.

Comparisons of SOD activities between oral-health behavior and attitude strata are shown in Table 2. Salivary SOD following scaling significantly increased from 83.09 to 194.30 U/g protein in patients who had irregular dental visit patterns (<1 visit per year).  Table 3 shows that the postscaling salivary TAOC was significantly higher in patients with a pattern of regular dental visits than in those with a pattern of irregular dental visits (3.52 versus 0.70 mmole/g protein; *P* < 0.01).

**Figure 2:**
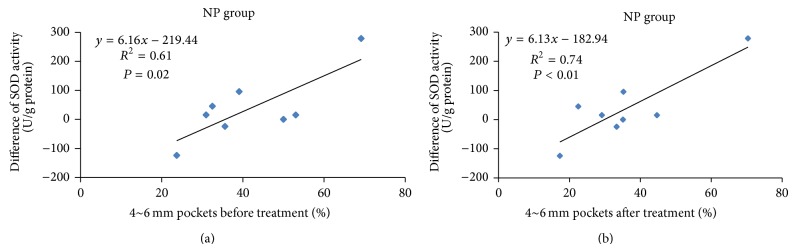
Scatterplots of superoxide dismutase (SOD) activity difference and 4~6 mm pocket percentages in the nonprogress (NP) group. (a) Percentages of 4~6 mm pockets before treatment. (b) Percentages of 4~6 mm pockets after treatment.

**Figure 3:**
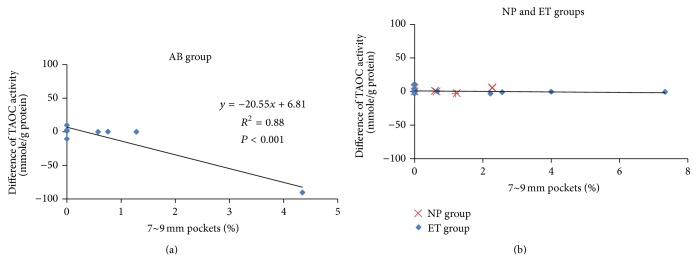
Scatterplots of total antioxidant capacity (TAOC) differences and sum of baseline 7~9 mm pocket percentages. (a) Abandoned (AB) group and (b) nonprogress (NP) group and effective treatment (ET) group.

Figure 2 shows scatterplots of SOD activity differences and 4~6 mm PD percentages in the NP group. In the NP group, SOD significantly increased with the percentage of 4~6 mm pockets before treatment (Figure 2(a), *R* ^2^ = 0.61, *P* = 0.02), and the same trend was also shown between the difference in SOD and completed treatment data of the percentage of 4~6 mm pockets (Figure 2(b), *R* ^2^ = 0.74, *P* < 0.01). The scaling-stimulated increase in SOD was more closely related to the severity of periodontitis in the NP group. The relationship between scaling-stimulated SOD and periodontitis severity was not significant in the other two groups. The TAOC significantly decreased from the baseline percentage of 7~9 mm pockets (*R* ^2^ = 0.88, *P* < 0.01) in the AB group (Figure 3(a)), but such an association was not seen in the ET or NP group (Figure 3(b)). The scaling-stimulated TAOC was significantly inversely related to the severity of periodontitis in the AB group.

## 4. Discussion

When an imbalance exists between bacterial species in the oral cavity and a host's immune response, periodontal disease may occur. [21]. ROS-mediated responses protect cells against oxidative stress and reestablish redox homeostasis [11]. Recent studies showed that nonsurgical periodontitis treatment is affected by salivary redox homeostasis biomarkers [14]. In this study, salivary SOD increased with an increase in the percentage of 4~6 mm PDs in the NP group, and the TAOC decreased with an increase in the percentage of 7~9 mm PDs in the AB group. These results imply that scaling-stimulated SOD or TAOC can be used as a biomarker for nonprogress or effective periodontal disease treatment.

Studies showed that SOD was significantly higher in pretreatment chronic periodontitis patients than in healthy controls [13, 22]. In healthy subjects, PDs increased with SOD (*r* = 0.46, *P* < 0.05) and the TAOC (*r* = 0.88, *P* < 0.05) [23]. Salivary SOD of pretreatment patients was significantly higher as the gingival index increased (*r* = 0.53, *P* = 0.02), but it was not associated with PD (*r* = 0.004, *P* = 0.99) [14]. The TAOC was also significantly higher in pretreatment chronic periodontitis patients than in healthy controls [22]. Another study by Brock et al. found that while the peripheral and salivary TAOCs were lower in periodontitis patients compared to healthy controls, the difference was only significant in plasma [24]. The oral cavity bacteria comprise one of the greatest discrepancies between periodontitis patients and healthy controls. The salivary antioxidant activity in rats infected with* Porphyromonas gingivalis* was significantly higher than that in a noninfected control group [23]. Controversial findings of antioxidants in periodontitis patients and healthy subjects in those studies may have been caused by the diversity of antioxidants in different biofluids and the virulence of periodontopathic bacteria.

As dental plaque harbors a number of bacterial pathogens which stimulate host cells to release various proinflammatory factors, the human body has developed an antioxidant defense system comprised of endogenous antioxidants to detoxify ROS and modify them to form less reactive species [12]. A study by Kim et al. showed that salivary SOD decreased after scaling and root planing therapy to 1 month but had increased again at 3 months in periodontitis patients [13]. Scaling stimulates gingival tissues. Proinflammatory factors are released when tissues are irritated, and SOD is secreted to compensate for the proinflammatory effects [25]. In order to investigate the relationship between scaling-stimulated (acute exposure) antioxidants and treatment efficiency, saliva was collected before treatment and immediately after patients had received ultrasonic scaling. The tissues may have had a more severe inflammatory response after acute scaling stimulation, and SOD may be more compensatory in patients who had a nonprogress treatment outcome. Our results showed that scaling-stimulated SOD was related to the severity of periodontitis in the NP group.

Scaling as a treatment for periodontal disease involves the thorough mechanical debridement of the dental calculus. In this study, all periodontitis patients received ultrasonic scaling. An* in vitro* study showed that stimulation by low-intensity pulsed ultrasound activated a cell growth signaling pathway in chondrocytes [18]. Low-intensity pulsed ultrasound also stimulated human circulating angiogenic cells to release endothelial nitric oxide synthase from 1 to 6 h in a time-dependent manner [19]. Most studies showed that the TAOC of periodontitis patients was lower than that of healthy controls [13, 14, 24, 26, 27]. The posttreatment TAOC was higher than that seen at the baseline in periodontitis patients [26]. Antioxidants help prevent cell damage by free radicals. The TAOC test is a measure of the endogenous (innate) and exogenous (food or vitamin intake) antioxidant capacities in a biological specimen [28]. Antioxidants are associated with the pathogenesis of a variety of inflammatory diseases and have a direct or indirect role in tissue repair [25]. The one relevant factor in AB group patients who discontinued their clinical treatment was periodontitis symptom relief. The relief may have been due to plaque having been eliminated and the TAOC being released after scaling irritation. Antioxidants may have been conserved in AB group patients and antioxidants were released after scaling stimulation. Our results showed that the scaling-stimulated TAOC was inversely related to the severity of periodontitis in the AB group.

Large-scale studies have shown that high standards of oral hygiene can prevent periodontal diseases and ensure the stability of periodontal tissue support [6]. Oral-hygiene-related behaviors also influence treatment outcomes with periodontal disease. Periodontitis patients who received oral-hygiene reinforcement during periodontal treatment had a greater probing PD reduction than patients who did not receive oral-hygiene instructions [29]. Plaque scores were significantly higher in periodontitis patients who had maintained regular dental check-ups than those in periodontitis patients who had not maintained regular dental check-ups [30]. Regular dental visits are related to the perceived severity of oral disease. The perception is related to personal oral hygiene and regular tooth brushing [16]. In this study, scaling-stimulated SOD significantly increased in patients who had an irregular dental visiting pattern. Patients who had an irregular dental visiting pattern may have had more dental plaque which caused inflammation in the oral cavity, and SOD may be more compensatory in those patients after scaling stimulation.

The postscaling TAOC of patients who regularly visited the dentist was significantly higher than that of patients who irregularly visited the dentist. Although there were no significant differences in the TAOC, tooth-brushing frequencies, or oral-health attitude scores, it was interesting that the postscaling TAOC was higher than that seen at the baseline in patients with good oral-health behaviors and attitudes (Table 3). In this study, patients who had a regular dental visiting pattern may also have perceived a greater severity of oral disease. These patients might intend to get more exogenous antioxidants from daily intake. The TAOC was more conserved in those patients, and scaling led to a significant induction of the TAOC.

Biomarkers in biofluids are useful tools for evaluating the activity of periodontal disease. Biomarkers in saliva, as well as in serum and plasma, are good indicators for use in studying periodontal disease [31]. Saliva is a biofluid that exists between local and systemic levels, and salivary antioxidants can give insights into both the oral cavity and systemic physical and pathological conditions. Analysis of saliva can provide access to molecular biomarkers of a variety of oral and systemic diseases and conditions, making it an ideal translational research tool [32]. These salivary biomarker detectors can be applied for periodontal disease screening and detection. An intracellular redox imbalance is associated with extracellular matrices and damage to their components. The biological effect of excess antioxidants in a redox state results in periodontal tissue repair. On the other hand, excessive ROS result in periodontal tissue damage when the redox state is imbalanced [25]. Scaling stimulates the gingiva, and saliva is an applicable biofluid to assess responses to local periodontal tissue changes. This is the first study to investigate scaling-stimulated local antioxidants as biomarkers for associations with oral health and periodontal disease treatment outcomes. For nonsurgical periodontitis patients, scaling-stimulated SOD may be a biomarker of poor treatment progress, while the scaling-stimulated TAOC may be a biomarker of effective treatment.

Since periodontal disease is largely preventable through lifestyle modifications, success in periodontal treatment is highly dependent upon personal oral hygiene. In this study, scaling-stimulated SOD increased in patients with irregular dental visiting patterns, and the scaling-stimulated SOD and TAOC were associated with the severity of periodontitis. These results demonstrate the importance of scaling-stimulated antioxidants as biomarkers of periodontal treatment prognosis. Our data implied that scaling-stimulated antioxidants may be influenced by oral-health behaviors and attitudes and may be sequentially related to treatment outcomes. In periodontal patients with large-scale changes of scaling-stimulated antioxidants, healthcare providers should seek to improve patients' oral-health behaviors in order to improve clinical treatment outcomes. Because of the sample size, we were unable to investigate the interaction between the scaling-stimulated antioxidants and oral-health behaviors in periodontitis treatment efficacy. Longitudinal data would be useful for testing causal pathways of oral-health behaviors and attitudes with acutely stimulated antioxidants in periodontitis treatment efficacy in the future.

## Figures and Tables

**Table 1:** Demographic characteristics, oral-health behaviors, and oral-hygiene attitude scores of study subjects.

Distribution of subjects	AB group (*N* = 8)	NP group (*N* = 8)	ET group (*N* = 14)	Fisher's exact test *P* value
	*n* (%)	*n* (%)	*n* (%)	
Gender				1.00
Male	3 (37.50)	3 (37.50)	6 (42.86)	
Female	5 (62.50)	5 (62.50)	8 (57.14)	
Education				1.00
High school	2 (25.00)	2 (25.00)	4 (28.57)	
University or above	6 (75.00)	6 (75.00)	10 (71.43)	
Smoking				0.59
Nonsmoker	5 (62.50)	7 (87.50)	9 (64.29)	
Smoker	3 (37.50)	1 (12.50)	5 (35.71)	
Alcohol consumption				0.77
Never or occasional	7 (87.50)	8 (100)	12 (85.71)	
Regular	1 (12.50)	0 (0.00)	2 (14.29)	
Betel nut chewing				0.48
Nonchewer	8 (100.00)	8 (100.00)	12 (85.71)	
Chewer	0 (0.00)	0 (0.00)	2 (14.29)	
Dental visit pattern				0.36
Regular visits (1 year)	5 (62.50)	5 (62.50)	12 (85.71)	
Irregular visits (≥1 year)	3 (37.50)	3 (37.50)	2 (14.29)	
Tooth cleaning frequency				0.16
<2 times/day	0 (0.00)	0 (0.00)	4 (28.57)	
≥2 times/day	8 (100.00)	8 (100.00)	10 (71.43)	
Oral-hygiene attitude score				0.71
<5 points	3 (37.50)	4 (50.00)	4 (28.57)	
≥5 points	5 (62.50)	4 (50.00)	10 (71.43)	

AB group: abandoned group (did not complete the entire program); NP group: nonprogress group (completed the entire program but treatment was not efficacious); ET group: effective treatment group.

**Table 2:** Comparison of superoxide dismutase (SOD) activity between oral-health behavior and attitude strata.

SOD activity (U/g protein)	*n*	Before scaling		After scaling		*P* value^a^
		Median	Q1–Q3	Median	Q1–Q3	
Dental visiting pattern						
Irregular visits (≥1 year)	8	83.09	51.04–100.15	194.30	101.64–274.85	0.02
Regular visits (<1 year)	22	75.44	49.22–208.86	98.79	60.44–246.38	0.81
*P* value^b^		0.38		0.31		
Tooth-brushing frequency						
<2 times/day	4	64.56	45.33–131.78	156.60	98.79–257.48	0.37
≥2 times/day	26	85.04	50.28–149.48	124.90	60.44–264.87	0.46
*P* value^b^		0.60		0.56		
Oral-health attitudes score						
<5 points	11	67.10	46.53–79.89	115.94	66.21–214.81	0.10
≥5 points	19	106.97	49.22–208.86	133.89	60.44–284.83	0.81
*P* value^b^		0.14		0.93		

^a^Wilcoxon signed-rank test.

^b^Mann-Whitney *U* test.

**Table 3:** Comparison of the total antioxidant capacity (TAOC) between oral-health behavior and attitude strata.

TAS activity (mmole/g protein)	*n*	Before scaling		After scaling		*P* value^a^
		Median	Q1–Q3	Median	Q1–Q3	
Dental visiting pattern						
Irregular visits (≥1 year)	8	3.06	1.61–4.97	0.70	0.00–2.78	0.21
Regular visits (<1 year)	22	3.06	1.65–7.67	3.52	0.83–8.46	0.38
*P* value^b^		0.68		<0.01		
Tooth-brushing frequency						
<2 times/day	4	6.06	3.05–8.43	3.05	1.06–6.51	0.12
≥2 times/day	26	3.06	1.70–4.97	4.13	0.96–9.03	0.42
*P* value^b^		0.35		0.13		
Oral-health attitudes score						
<5 points	11	4.23	1.70–6.37	3.81	0.69–13.81	0.89
≥5 points	19	2.99	1.61–7.67	4.46	1.05–8.34	0.97
*P* value^b^		0.45		0.54		

^a^Wilcoxon signed-rank test.

^b^Mann-Whitney *U* test.
